# Thalassemia, biobanking infrastructures, and personalized stem cell therapies in Chennai

**DOI:** 10.3389/fsoc.2023.1057220

**Published:** 2023-07-27

**Authors:** Amishi Panwar

**Affiliations:** Department of Population Health Sciences, Bristol Medical School, University of Bristol, Bristol, United Kingdom

**Keywords:** biobanks, Chennai, cord blood, ethnicity, HLA, personalized therapy, thalassemia, stem cells

## Abstract

Thalassemia and leukemia and related blood disorders are approved for blood stem cell transplants in India, for a stem cell transplant to be successful, the human leukocyte antigen (HLA) complex located on the arm of chromosome six must be a match between the cord blood donor and the recipient. In the quest to find an exact blood stem cell match for an individual, the HLA becomes the node at the center of community genetics where the HLA match is sought (not necessarily successful) in the extended family, the same caste, language, and ethnic (both national and the diaspora) groups. By considering thalassemia as a case study, how do we understand personalized stem cell therapies within biobanking infrastructures in Chennai? How do social categories get entwined with biological materials like cord blood?

## Introduction

Biobanks come in various types (a) to cater to public needs (Gottweis and Lauss, 2011; Beltrame and Hauskeller, 2016, 2018), (b) for private or family use (Santoro, 2011), or (c) for community use. Tissue samples with respect to stem cells include blood (Starr, 2002), cord blood (Wagner and Gluckman, 2010), menstrual blood (Fannin, 2013), peripheral blood, and dental pulp (Collart-Dutilleul et al., 2015). Cord blood stored in biobanks is a potential source for hematopoietic (blood-producing) stem cells. These cells can regenerate the entire blood system (Cooper and Waldby, 2014) in severe cases of blood disorders such as thalassemia (a genetic blood disorder) and leukemia (blood cancer). Cord blood becomes significant because blood stem cells extracted for a stem cell transplant are important for categorizing HLA, thereby finding a match for a stem cell transplant.

Each cell in the human body consists of threadlike structures called chromosomes^1^, which consist of intertwined strands of DNA (deoxyribonucleic acid: the repository of genetic information). The HLA complex is located on the short arm of chromosome six. HLA antigens are glycoproteins that reside on the surface of almost every cell in the body. The primary function of these antigens is to serve as recognition molecules in the initiation of an immune response (Scaradavou, 2013). HLA is a critical component in establishing a match between a donor and a recipient. Once a match is established, a (stem) cell transplant can take place. Cord blood stem cells are preferred because they are “young” (Alvarez-Palomo et al., 2019) “raw, new and adapt easily (from personal interaction with a cord blood bank stakeholder at a conference in New Delhi, January 2017).” If a perfect match is not found among the parents and siblings (or anyone related), an unrelated donor is sought, making public storage of cord blood units a preferred choice among transplant physicians and the biobanks.

Considering HLA as data stored against categories of individuals understood as belonging to a family, caste, language, or race, I use Pinel and Svendsen's (2021) definition of personalized medicine. Personalized medicine is seen as a flow of data from the “personal” (data representing individuals) to the “collective” (common resources representing the population) and then to the “personalized,” i.e., representing populations but stored for individual benefits (p. 2), therapy, and recovery. I build this article as follows: The first part explores thalassemia as a case study where the cord blood unit can be seen at the center of both the social and biological. Given the high incidence of thalassemia in South India, how do biobanking infrastructures help in finding an exact HLA match for stem cell therapy and treatment? Over time, HLA matches have been likened to the “collective” representation of populations where cord blood samples are stored for certain social categories of populations. Therefore, caste, race, and language become seen as communities catering to making therapy “personal.” Therefore, in this article, I investigate, how do cord blood banking infrastructures help in finding personalized HLA match to treat blood disorders like thalassemia. How do social categories get entwined with biological materials like cord blood?

## Materials and methods

I was a Research Assistant in India with a project titled “The Red Revolution: Emergence of Stem Cell Biotechnologies in India” based at the Geneva Graduate Institute, Switzerland and funded by the European Research Council (ERC) from 2014 to 2015. My fieldwork was supported by this project from 2015 to 2017. The study involved researching and archiving media reports, managing data and documenting, identifying available gray literature, writing reports, collecting and annotating relevant publications, and scoping the field for stem cell facilities in India. By stem cell facilities, I mean transplant and research centers, banks and their subsidiaries, and governing bodies in India. Among these, stem cell facilities were numerous private cord blood banks and a number of public cord blood banks. In India, umbilical cord blood stem cells are used to treat thalassemia, leukemia, and related blood disorders, whereas cord blood stem cell treatment for all other disorders is classified under research and requires registration of a clinical trial with the governing body in India.

Once I compiled a list of facilities in India, it was clear that the largest public cord blood bank was in Chennai. Chennai is also the headquarters to major private cord blood banks and home to the largest blood stem cell registry in India. Most of these banks are networked with the blood and marrow transplantation center, Apollo Hospitals in Chennai, which has performed over 1,500 stem cell transplants (as of September 2022) with a high success rate. I conducted 20 interviews with stem cell bank owners, scientists, doctors, technicians, marketing agents, and counselors with limited and difficult access given the secrecy surrounding the stem cell industry in India−15 involved semi-structured open-ended interviews and limited observation at cord blood banks. I also interviewed 60 people at a maternity clinic in Chennai, including pregnant women and their respective families, initially with a semi-structured open-ended interview questionnaire, but later switched to having conversations with them in the waiting room. Through the prewritten interview questions, my intention was to survey the number of women and their families opting for cord blood banking, be it public, private, or community banking (Panwar, 2023). I established contact via email with LifeCell (a private cord blood bank), Jeevan (a public cord blood bank), and W, a blood stem cell registry in Chennai. LifeCell is one of the largest operational private cord blood banks in India. It began operations in 2004. It has about 375,000 units of cord blood units banked (as of July 2023), having started as a technological partner with Cryo-Cell International, the world's first private stem cell bank based in the USA. With over 200 centers spread across India, LifeCell is headquartered in Chennai and has a fully functional cord blood processing facility in the suburbs of Chennai and Gurgaon in the National Capital Region of Delhi.

Jeevan cord blood bank began as a blood bank initially. Later as a a public stem cell bank (encouraging voluntary donations of stem cells), it started operations on principles like the blood bank with the help of corporate sponsors, philanthropists, and community support. I first met Dr. Srinivasan in the summer of 2015 in his office located in a building that housed a blood stem cell laboratory and bank. He has published widely with his colleague and co-founder of Jeevan, Dr. Saranya Narayan, on the need for cord blood storage facilities in India, voluntary donations, and the necessity of HLA-matched donors for a diverse country like India. Dr. Narayan introduced me to Dr. X at Apollo Hospitals in Chennai, a leading pediatric oncologist in Chennai. To work with Dr. X, ethics approval was required from the Ethics Committee at Apollo Hospitals in Chennai, the largest private hospital in South India. Having obtained the clearance from the Committee, I was invited by Dr. X to the hospital's children's wing. Dr. X made it clear that I would not be allowed to interact with the patients saying that it would not be right to ask the parents anything as they were not in a state of mind to respond. I agreed to observe and interact with junior doctors about stem cell transplants and related procedures. Similarly, none of the biobanks allowed interaction with patients, clients, donors, and recipients citing confidentiality as a reason. At Jeevan cord blood bank, I observed sorting, labeling, and processing of seven cord blood units with the help of laboratory staff. While at LifeCell, I was only allowed interviews with the scientific director and the marketing office; the processing of cord blood units happened behind closed doors.

This research has largely been multi-sited (Marcus, 1995; treating multi-sited ethnography as a conceptual topology requiring different methodological strategies, access to a different range of sources, and different narrative strategies), considering different sites of observation in Chennai and New Delhi between 2015 and 2017. All interviews were recorded with permission and a signed consent form (signed by myself and the interviewee at the end of the interview). Most of my interlocutors' names in this article are anonymized, and in cases where they are identified, it is with permission on a signed consent form. I performed all Hindi-to-English language translations. Patient anonymity has been maintained, and data, documents, and information arising out of this research are confidential, i.e., only subject to my research and analysis. The study has been conducted as per the prevailing ethical guidelines of the Geneva Graduate Institute and ethical committee approvals obtained with concerned hospitals and biobanks in India. Data collection, analysis, and writing have been performed simultaneously to prevent any loss of substantial information and to draw valuable insights and inferences continuously throughout the course of the research.

### Thalassemia, cord blood, and stem cell matches

Across from me on the operating table at Apollo Hospitals in Chennai (2015) was a boy, aged 6. Dr. X walked in, as was routine, continued expertly to turn the boy onto his stomach and enquired about the right dosage of anesthesia. I watched as she injected a thick needle into the boy's hip. The needle did not pierce the flesh easily. She winced a bit, tightened her grip, and turned the needle a little to the left and a little to the right, almost drilling through the flesh. The boy moaned a little. She continued this process until the needle touched his hip bone. Her assistant immediately brought the collection bag and placed it on a steel table near the feet of the boy. The plastic sheet around the boy's hip collected the blood dripping from the fresh wound and the rest was transferred to the collection bag, continuously shaken to prevent clotting.

She turned to me as the child was turned onto his back, and the wound was cleaned and closed with white gauze. “This boy is saving his brother (aged seven-and-a-half) today. His brother is a thalassemic, and his sibling was found to be an exact match.” In this case, the sibling could be the donor, but in other cases, the quest for finding an exact match for a blood stem cell transplant sometimes took months or years (Panwar, 2020). This search for an exact stem cell match involves hospitals, banks, and registries—both national and international. This cocktail was essential as stem cells used for transplant need to match the patient's weight, and the ones collected separately by banks may not have had enough blood stem cells. Dr. X later added that in some instances the team injects a cocktail of stem cells sourced from various banks and registries—Jeevan (the public bank); LifeCell (the private bank); and W, a blood stem cell registry based in Chennai. This cocktail, she emphasized, was essential as stem cells used for transplant need to match the patient's weight, and the ones collected separately by banks may not have had enough blood stem cells. But blood, as we know, acquires meanings in its myriad states—fluid, solid, and viscous—and “becomes” (Copeman, 2009; Bennett, 2010) in every association it makes. Weston considered blood as having a “meta-materiality” (Weston, 2013; p. 35) blood's different evocations and imagery exist beyond its materiality and can still comment on each other (Copeman, 2014; cited in Carsten, 2019). Taylor (1992) explored blood as a liquid gift, a concept made popular by Copeman (2009) in his detailed ethnography about blood donation as a sacrifice and gathering merit (or *punya*, in Sanskrit). Therefore, how do we understand cord blood in relation to disease and biobanks? In movement and practice, how does the cord blood unit entangle social lives and biological material (Nading, 2017), thereby defining present-day biobanking infrastructures in Chennai?

Thalassemia is the most common inherited blood disorder across the world. It is estimated that about 100,000 children are born every year with blood-transfusion-dependent thalassemia and 1.1 percent of couples are at risk of having an affected child (Black et al., 2010; Bandyopadhyay et al., 2013; Panja et al., 2017). Previously known as the “Mediterranean disease,” the first recorded case of thalassemia East of the Suez was reported in 1938 (Mukherji, 1938; Verma et al., 2011). The word thalassemia, or sea in the blood, was coined by Nobel-prize-winning pathologist George Whipple and William Bradford: *thalassa* in Greek means “the sea” (like the Mediterranean Sea) + -*emia* means “in the blood.”^2^ Chattopadhyay (2006; p. 2662) showed that most of the studies on thalassemia have focused on “immigrant populations living in Europe or in the Mediterranean region” with others focusing on prevention, counseling, and screening in Iran (Samavat and Modell, 2004; Strauss, 2009), Thailand (Hartwell et al., 2004), Saudi Arabia (Al- Hamdan, 2007), Jordan (Hamamy et al., 2007), Iraq (Al-Allawi and Al-Dousky, 2010), Bahrain (Al-Arrayed, 2005), Turkey (Mendilcioglu et al., 2011), and Pakistan (Ahmed et al., 2002; Ishaq et al., 2012). Most of these studies have focused on pre-marital counseling and prevention given the high incidences of cross-cousin marriages. Populations of North Africa, West Asia, and South India prefer consanguineous marriages or the “coming together of blood” (Clarke and Parsons, 1997; p. 7) with 20–50% of these marriages being culturally and socially preferred and 33% of which are first-cousin unions (Tadmouri et al., 2009; Bittles, 2011; Hamamy, 2012). Proctor and Smith (in Clarke and Parsons, 1997; p. 98) suggest that birth outcomes are affected by behavioral factors (e.g., diet), environmental factors (e.g., poverty), healthcare services (e.g., equity of access), and finally genetic, consanguinity factors, and geography (Akinyanju in Clarke and Parsons, 1997; p. 133).

Medically, cross-cousin marriages in South India have led to various cases of genetic conditions, especially where the birth of the male child is deemed more important than aborting a child with thalassemia. Therefore, lifelong transfusions of red blood cells are considered better than not having a male child at all (from personal interaction with a transplant physician at Apollo Hospitals, 2015). The prevalence of cross-cousin marriages in South India has led to inherited blood disorders like thalassemia, the cure for which is a blood stem cell transplant sourced from banked cord blood. By introducing and discussing consanguineous marriages while counseling parents opting for cord blood banking, the natural fact of procreation is combined with the social fact of marriage and property and becomes part of a scientific discourse where kinship is medicalized (Strathern, 1992; Atkinson et al., 2001; Finkler, 2001). Linking genetics to disease rests on the premise of the medicalization of family and kinship. Genetic diagnostics is based on charting the family medical history and includes questions on inheritable diseases, marriage patterns, and working on finding the exact HLA match for a stem cell transplant. Both Schneider (1980) and Trawick (1992) have suggested that biogenetic kinship relations are bound by love, marriage, and choice. But biobanks take it a step further. It expands this circle of kinship and connects individuals and families via banking and voluntary donor networks through a community.

### Understanding biobanking infrastructures: *community* in personalized therapy

How does one find an exact HLA match for a blood stem cell transplant in cases of blood disorders? Over many conversations with the various stakeholders, both public and private, it was clear that the first criterion that a transplant physician will look for is an almost-exact HLA match. In a conversation with Mr. Y at W, the blood stem cell registry in Chennai:

[…] there is less than 25 percent chance of finding an exact match within the family. We don't say it, but we find members within the same caste. The markers are the same. For example, Rajalakshmi in London died this morning. We organized a drive for her but couldn't find a match. But you know it is necessary that you find an exact match within the same community. Of course, there are people who have got an Italian match. The donor is Italian, but the patient is Telugu speaking… Andhra… but the first preference is to look for matches within the community. So, Raji, for example, didn't find a match within the community.

As Rajalakshmi (name changed) was from Andhra Pradesh in South India, the search for a match began with her family and moved outwards to the joint family, her caste members (details were not shared with me keeping in mind client confidentiality), and finally, the search was expanded to the World Marrow Donor Registry.^3^ In addition, caste and community are being used interchangeably in this conversation with Mr. Y. Although searches for a stem cell transplant involve creating a fully functional registry where any person in need (in India) can find a match, the word “community” has been used as an umbrella term for members of the same caste, ethnicity, race, and related family members. A member of the marketing team at LifeCell stressed that community means family for the time being:

“So when the child's stem cells come into the community, father also gets access to the entire community. Father, mother, and future siblings are the community, these four people can access the stem cells from the community. I think in a few years, even cousins can get access. So you can mix samples from the community and use them if the quantity is not enough.”

Brown and Kraft (2006; p. 321–323) highlight that even though the banked blood might not be an exact immunological match within the family, it is presented as a “family asset” and these become “communities of promise.” Santoro (2011; p. 86), following Brown and Kraft, explains that “the familial body is projected in new ways toward the future and the past: toward an act of responsibility over expectations of biotechnological futures, and toward a past of familial “genetic” diseases—real or imagined—that is often behind the decision to bank the cord of one's child.”

**Figure 1:**
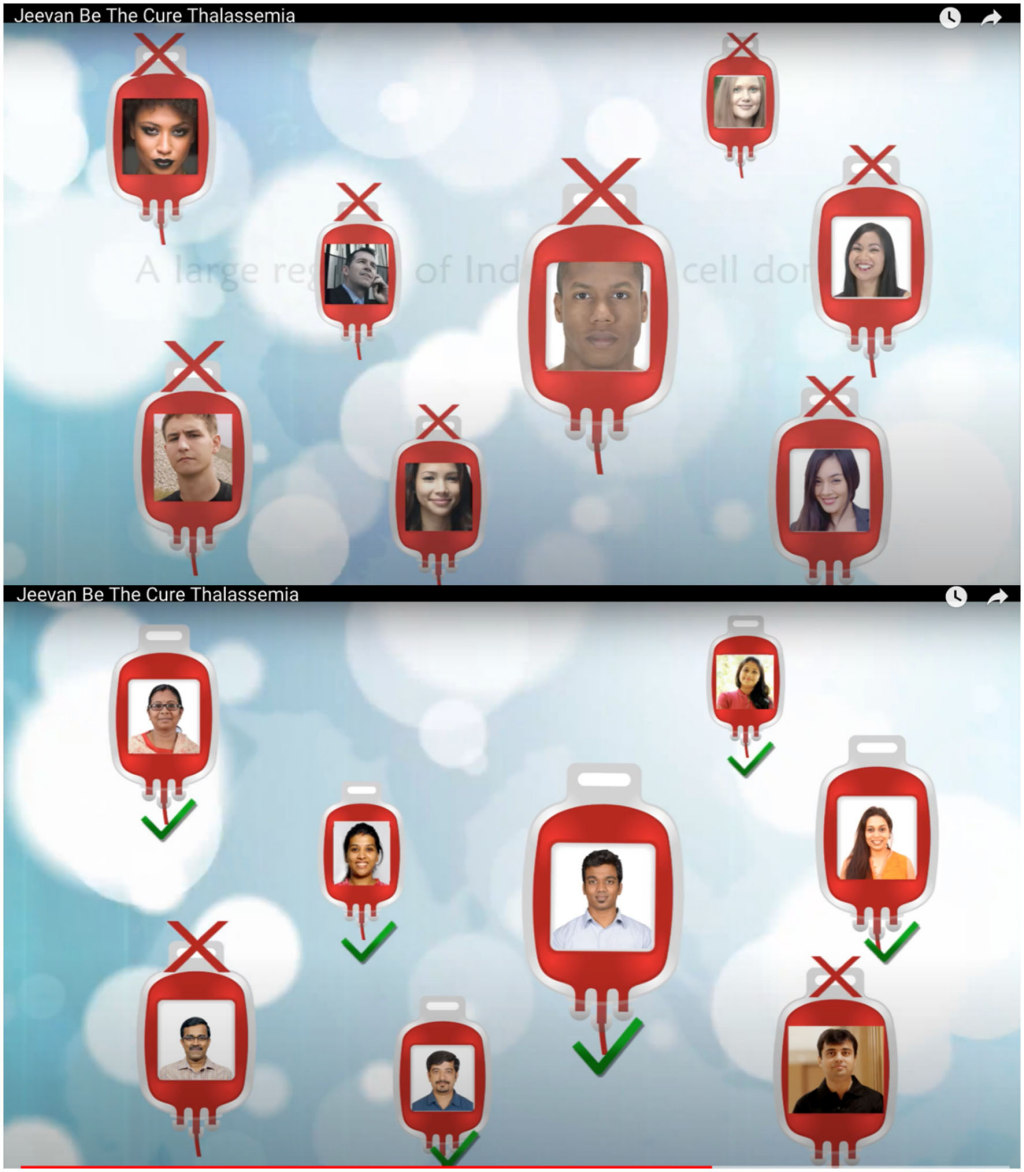
An initiative to encourage Indians to donate stem cells with the aim of building a national registry.^4^

Moreover, since my first meeting with Dr. Srinivasan and Dr. Narayan at Jeevan in 2015, they were clear about the benefits of voluntary donation of cord blood and emphasized HLA matches being ethnicity specific. In a 2018 article, matches being ethnicity dependent was clarified further: “HLAs are ethnicity dependent. With the poor representation of the potential donors of Asian origin in the database of over 32 million donors listed by the World Marrow Donor Association (WMDA), the chances of an Asian finding a 10 out of 10 HLA match for HSC (Hematopoietic Stem Cell) transplantation are very low. Consequently, hope for [sic]potential cure through transplantation is far lower for Asian patients compared to Caucasian patients” (Periathiruvadi, 2018; p. 5). In March 2017, Jeevan released a short movie on YouTube highlighting the need for voluntary blood stem cell donations for public use (see Figure 1).

These two images are screenshots taken from the short movie made and advertised by Jeevan to create awareness about donating cord blood and creating a registry. “For Indians and By Indians.” I was struck by the change of photographs from different races to photographs with people who visibly looked Indian in the second image. In advertising ethnicity as the source of finding an exact HLA match, science also calls for an affinity and similarity (Bärnreuther, 2018) among blood relatives (Street, 2009). It also suggests that genetics provides a language of kinship and affinity, creating communities of individuals banking on genetic advancements, risk, and research (Silverman, 2008). “Folk notions of [sic]family as a biogenetic entity allow for an effortless embrace of the scientific and biomedical notion of genetic determinism *precisely because it mimics cultural conceptualizations of the biogenetic foundations of kinship*” (Finkler, 2001; p. 247). By projecting cord blood as a unit capable of treating members of a family upon a complete or partial HLA match for a stem cell transplant, cord blood banks rely on the popular cultural notion of “parental responsibility, family ties, and private property” (Brown and Kraft, 2006; p. 325), thereby extending the circle of non-related donors to create a community of parents banking cord blood. In this case, the concept of community cord blood banking depends on the family as a biogenetic entity and draws on the concept of kinship with its basis in family and blood ties. Therefore, the first beneficiary of the cord blood unit is the child whose blood is banked. The cord blood unit then belongs to family members if an exact match is established and, finally, to the community of people who have come together to invest in the processing and storage of the cord blood unit.

Mauss' (1990) concept of the gift relation, that voluntary giving and receiving will promote reciprocity and promote a collective/community understanding, holds true in this context. The concept of voluntary blood donation having qualities of civil inclusion and social justice was first introduced by Titmuss (1997). In an era of neoliberalism and globalization, where the collective (i.e., community) seems bleak (Waldby, 2006), Jeevan is working toward the opposite, i.e., establishing a community of people who donate voluntarily. Instead of “wasting precious cord blood,” why not donate (reads the flyer promoting public cord blood banking at Jeevan, March 2017)? Moreover, the updated National Stem Cell Guidelines in India 2017 states:

Public cord blood banks across the world, for several decades, are playing an important role as a source of HSCs for transplant in selected hematological conditions. Hence, parents should be encouraged for voluntary donation to public cord blood banks for allogeneic use based on HLA matching and for research purposes. Obstetricians must educate parents to be, about the options available, especially donating cord blood to a public bank (National Guidelines for Stem Cell Research (NGSCRT), 2017, p. 37).

Waldby further states that voluntary donations (of cord blood) can be treated as a “compromise position between the social generosity of the gift and the exploitative utilitarianism of tissue markets. It involves neither a generous donation nor an exploitative acquisition, but rather uses the individual's body as its own resource, potentiated by prosthetic or ex-vivo intervention” (Waldby, 2006; p. 59). But are voluntary donations of cord blood easy in a country like India where blood (read cord blood) is seen as a marker of social categories like caste and family as a biogenetic unit?

### Understanding biobanking infrastructures: *South Asian* in personalized therapy

A conversation with Mr. Z, area head of sales at LifeCell, presented clarity to the case at hand. Mr. Z has been in the cord blood industry for over a decade. I asked him whether people have problems donating cord blood. My curiosity stems from the fact that blood donation is often associated with the blurring of caste and religious boundaries in India (Copeman, 2009).

I … you know when parents find out that you cannot use it for your own child… then they won't agree…. You know it's like their own possession. So first they say yes (referring to cord blood donation/public banking) and then *no no humko karne do…* (no no let us do it) referring to private banking this is what we have seen plus the criteria (referring to the checklist for a cord blood unit to be banked) for banking in public banking is high and that's the reason only 50-50 samples are in the public bank. …. India… it is a HLA diverse country… getting a match is so difficult… there are castes, creed, and [also] marriages within the community (referring to consanguineous marriages) […] Here, we have Asian but in community banking… you get an ethnic match…

The need for Asian donors or the claim that the Asian genetic makeup has its roots in the study of populations for biomedical research, as Tupasela points out, draws on historico-cultural narratives of national genetic heterogeneity to brand themselves as ideal targeted populations for medical research (Tupasela and Tarkkala, 2018; p. 741). The branding of genetic uniqueness for population research has been seen in various studies based in Iceland (Rose, 2001; Pálsson, 2007; Fortun, 2008), India (Sunder Rajan, 2006), Israel (Prainsack, 2007), Mexico (Kent et al., 2015), Brazil (Gibbon, 2013), Singapore (Ong, 2016), and Estonia (Fletcher, 2004). This is also linked to the marketability and advertisement content of biobanking practices, thereby addressing questions of inclusion and identity (Tupasela and Tarkkala, 2018; p. 741–743). This embodiment of a need (e.g., being healthy) in the other/the outside gives rise to what he calls the “social imaginary,” the individual projection of one's hope, fear, and anticipation (Bennett, 1976; p. 848) in a larger moral domain of biobanking practices and stem cell transplant. Therefore, when a family decides to bank their child's cord blood, be it in private banks, or donated to public banks, the decision is built on the anticipation of disease and fear of ill health combined with the hope of being cured.

**Figure 2:**
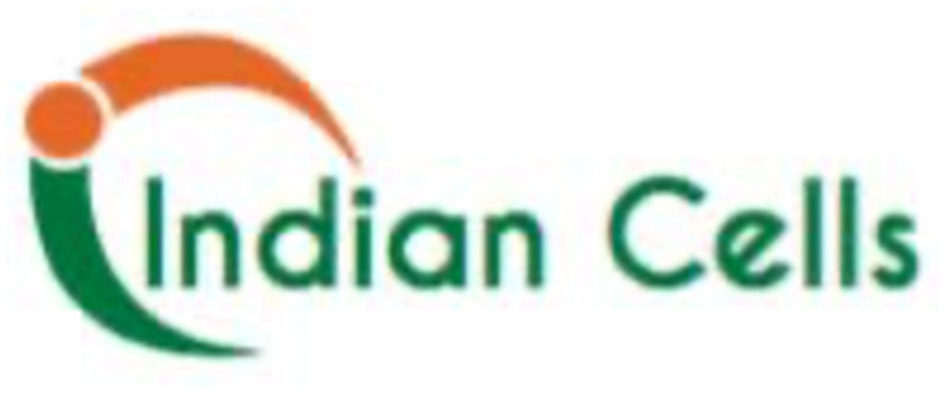
This figure of “Indian cells” has colors from the Indian national flag: saffron, green, and white. It represents Jeevan's campaign to have a “million cells” (stem cells) for Indian patients in need. (Source: https://www.indiancells.in/).

The need for Asian donors in registries across the world was brought to the fore when Nalini Ambady, Professor of Psychology at Stanford University lost her life to leukemia in 2013 after a year-long battle. This case was cited by most of my interlocutors at LifeCell as the turning point for investing in Asian- and India-specific donor registries, thereby investing in community banking. Experts had suggested a bone marrow transplant and a perfect 10/10 HLA match for a transplant to save her life as both her children were not a perfect match. A campaign was launched by her daughter to find a perfect match; 12 donors were found, of which six were ruled out as the HLA match was not perfect. Once the 6/6 match was obtained, doctors proceeded to the 10/10 HLA match; six of the 12 potential donors were a match. All but one backed out, and he was from Professor Ambady's home state in Kerala, South India. Unfortunately, the final donor was talked out of the procedure by his parents. The conflation of race (i.e., Asian)^5^, ethnicity, and community is further complicated by introducing the very recent “Indian cells” and language-specific registries (both launched by Jeevan 2017, see Figure 1 for one such media initiative). While “Indian cells” has a nationalistic fervor in its establishment (with colors of the national flag in its logo, see Figure 2), the need is also scientifically valid as the case of Professor Ambady shows.

In an interview with *The Hindu*, a leading national daily, Dr. Srinivasan said, “Our goal is to have 30,000 donated cord blood units and 1,00,000 bone marrow donors registered by 2022. This will enable 70 per cent of Indians with blood cancers to find a match and hope for a cure” (Hamid, 2017). While Dr. Narayan, the co-founder of Jeevan added, “This is truly a Make in India^6^ project.” Be the Cure registry, run by Jeevan Stem Cell Foundation, is the largest repository of stem cells obtained from donated cord blood in South Asia and a fully functioning adult marrow donor registry.^7^ Dr. Srinivasan, in conversation with *The Hindu*, mentioned the grant given by the government of Tamil Nadu.

Every year, 10,000 children are born with thalassemia and every year over a lakh people are identified with leukemia. If they were to look for an HLA match for treatment, there is no inventory of any great size in India yet. An HLA match is ethnicity dependent. When an Indian is looking for a match, there is a greater likelihood of finding a match within an Indian inventory. In 2013,^8^Tamil Nadu Government was the first government to realize the importance of this programme and has given us funding to the tune of INR nine crores (~USD 140,000) spread across three years (Hamid, 2017).

The aim as Dr. Srinivasan suggests is to establish the largest registry for Indian stem cell matches in South Asia. Health is a state subject^8^ in India, and Tamil Nadu has outperformed all other states in India on specific social development indicators, such as primary education and health. The state government, therefore, supports subsidies and has adopted a social spending approach in matters concerning health (Menabde, 2015). *The Hindustan Times*, another leading daily, reported that a consortium of blood stem cell registries is required in India to make a national donor registry and in turn, maintain uniform standards and cost of treatment (Kaul, 2019). The term “national stem cell registry^9^,” as we have seen, is being touted as a consortium of state and language-specific registries, which will combine to make the national stem cell donor registry.

### Understanding biobanking infrastructures: *Tamil* in personalized therapy

Dr. Srinivasan: “[…] The first thing (for a stem cell transplant) is the HLA match, [sic]the second is the blood group. Nobody looks at religion, caste, or language. But it does come from the point of view of [sic]registry. India has twelve major languages…from the HLA point of it and the haplo point of it, nine major linguistic groups have their own minor variation. In fact, we have submitted the publication^10^… for Tamil speaking population and the next is Urdu, Hindi, and Malayalam. This is the basis on[sic] the govt of Tamil Nadu's grant to us for the 3,000 samples…. They said we need it for the Tamil speaking population. And we collected [the samples] with the money provided and it will be added to the registry. This is science-based, not the politics of Tamil Nadu or Tamil story […] And if you do that for every state, then you have created a national registry in no time.”^11^

The politics of Tamil Nadu is worth mentioning as it is intrinsically linked to being Tamil and identifying as one. The Dravidian movement recognized the linguistic divide between the Indo-Aryan languages (North Indian, West Indian, and East Indian) and the Dravidian languages (South Indian). Sanskrit was considered a sacred language of the Aryan group, and the Brahmins who belonged to the upper caste had access to this language in practice. The divide was set in a manner that made the Dravidian language inferior, and the divide was made more prominent given the political dominance of the Brahmins under the Madras Presidency. As Venkatraman (2017) notes, anti-Hindi agitations lasted three years until 1940 till the move was repealed and rose again in 1950 when the Constitution of India was being framed. The opposition against Hindi being made the official language of the country by the national government continues even today and was also a matter of concern in the 2014 general elections. This need for difference, being separate from the “Hindi speaking North India,”^12^ has a regional nationalistic fervor to it and has been so for a very long time in Tamil Nadu.

In 2015, When I sat in the office of Jeevan in Chennai listening to Dr. Srinivasan narrate his story, he suddenly stopped and asked, “you tell me, how can I include the diaspora in expanding this bank? [pause] You know I have to create a Tamil repository” (field notes, July 2015). The statement had a flavor of pride in it and is stated rather nonchalantly. Tamil is a language, an identity, and a political tool used to build an ethnic community separate from the rest of India. Tamils have lived in the Southern portion of peninsular India for over 3,000 years, and today, most of them live in Tamil Nadu. Tamil belongs to the Dravidian family of languages along with Telugu, Kannada, and Malayalam. It is significant to note that “Tamils alone have remained culturally and linguistically least influenced by the Sanskritic civilization of North India” (Pandian, 1998; p. 546).

**Figure 3:**
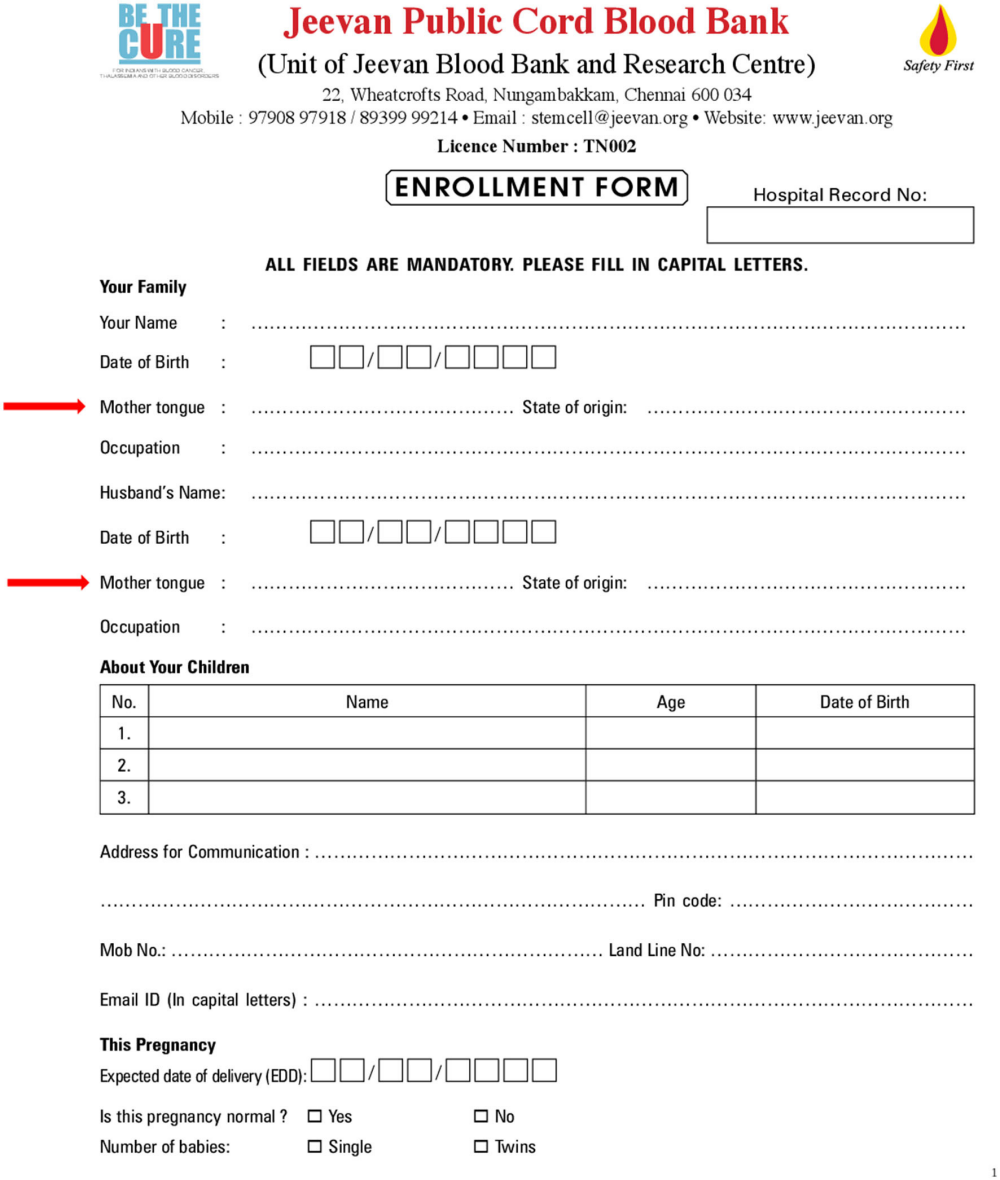
Enrollment form of Jeevan stem cell bank (red arrows added). Data on parents' mother tongue/native language collected to match inherited haplotype frequencies in the Tamil-speaking population to make a Tamil registry available for recipients (Scanned document by author, August 2017).

The mother tongue or one's native language is therefore used as a marker against the donors' sample, making a language-specific registry possible (see Figure 3). A 2018 study with data obtained from donated cord blood units at Jeevan identifies certain inherited haplotypes (set of genetic determinants inherited from both parents)^13^ frequencies in the Tamil-speaking population (Narayan, 2018). A recent study proposes a link between South Indian language groups (Malayalam, Urdu, Kannada, Telugu, and Tamil) and finding donors for patients based in Sri Lanka (Seshasubramanian et al., 2021). Hegde mathematically posits the link between HLA and language and proves the relationship using Nei's genetic distance formula. Laboratory work for this thesis was carried out at *Jeenomics*, the HLA laboratory at Jeevan. The aim of Hegde's work was to build a first-of-a-kind (in India) population-based genetic model for four native language groups: Tamil, Telugu, Hindi, and Urdu. HLA data for this were obtained from the UCB registry at Jeevan.

A study by Chen et al. (1995; p. 607) placed geographic distance as a major confounding factor in the correlation between genetics and linguistics. Their study is based on the fact that languages change over geographic space, possess numerous characteristics, and have a phylogenetic (the evolution of a genetically related group of organisms as distinguished from the development of the individual organism^14^) history. Another study by Cavalli-Sforza et al. (1992; p. 5621) found that populations speaking languages from the same family tend to be genetically related. For example, the Indian population is grouped under the Indo-European language group, and the Southeast Indian population is grouped under the Dravidian language group. Nei explains genetic distance as a “statistical method for estimating the number of codon differences per gene and the divergence time between closely related species. This method utilizes electrophoretic data on protein identity between different species (Nei, 1972; p. 283). Thus, the average number of codon or nucleotide differences per gene is a measure of genetic distance […] when two species to be compared are distantly related, data on amino acid or nucleotide sequences are used” (Nei and Kumar, 2000; p. 828). Hegde calculated pairwise Nei's genetic distance on each of the four native language subgroups (Tamil, Telugu, Urdu, and Hindi) and arrived at the following:

The results state “lesser the Nei's genetic distance value, [sic]greater is the population related to each other.” Table 1 clearly shows that the population with Tamil as their native language is most related to the population with Telugu as their native language, and vice versa. The population with Urdu as their native language is most related to the population with Hindi and Tamil as their native language (Hegde, 2018).

**Table 1:** The proximity of languages in South India based on genetic distance (Source: Hegde, 2018).

**Native language**	**Tamil**	**Telugu**	**Urdu**	**Hindi**
Tamil	0.0000	0.0361	0.0522	0.1700
Telugu	0.0361	0.0000	0.0563	0.1582
Urdu	0.0522	0.0563	0.0000	0.1494
Hindi	0.1700	0.1582	0.1494	0.0000

The point Dr. Srinivasan and Hegde make is that finding HLA matches within a certain language subgroup is higher and India needs to build a registry for each of these language subgroups. Lupton (1994) would have drawn our attention toward the “geneticization” of race or what Callon et al. (2005) have termed an “economy of qualities” where banks are deemed as repositories of a certain community of people. Only that in India and Chennai, we are not dealing with race but with ethnic groups deemed as language-specific communities. Today, much of the public cord blood sector is aimed at collecting cord blood of rare immunological types—particularly ethnic minorities for whom it is difficult to find a conventional donor (Brown and Kraft, 2006). Rabinow and Rose (2006) have introduced concepts, such as biosociality, where individuals and communities manage life by considering genetic risk and prevention. Ong (2016) provides an atlas of genetic mapping of cancers and their intervention in her study of Asian populations in Singapore. She explains that diagnosing cancers today focuses on genetic and protein markers in certain populations that are termed biomarkers. These biomarkers are acquired both epigenetically and genetically—the latter is where characteristics are acquired somatically, and the former is a result of living in a certain cultural lifeworld.

As Dr. Srinivasan said, “chances of finding my haplotype is[sic] higher among Indian donors and the success rate is likely to be higher from my own lineage: Tamil population.” Lineage is descent traced from either of the parents—either matrilineal (from mother) or patrilineal (from father). As Ong suggests, finding a genotypically similar match is closely linked to the “ethnicity heuristic” and is a more “sophisticated way of marketing science” (Ong, 2016; p. 30–31). In the case of cord blood banks and unrelated donors in Chennai, the biomarker is being termed as the language, Tamil. By analyzing the HLA frequencies of parents (who donate their child's cord blood and mark Tamil as their mother tongue), the final aim, as Dr. Srinivasan suggests, is establishing the national stem cell donor registry. By locating Tamil as a biomarker, Jeevan stem cell bank establishes the first step to finding a match, and this, as most of my interlocutors suggest, is based on finding positive matches within the same ethnic community.

## Conclusion

Banking on the cord blood community, therefore, becomes a “life strategy” where genetic endowment (Novas and Rose, 2010; p. 488) is considered as both the diagnostic and the cure.

“[And] by deploying ethnicity as a code for identifying specific genetic risks and mutations, […] the goal is to customize the right cocktail of drugs for a particular patient so that the disease can be managed as a chronic condition. Nevertheless, *by zeroing in on ethnic, family, and individual genetic targets, the new research milieu is also productive of affects of fear, hope, and pride in a novel form of scientific self-knowledge, one that frames “personalized medicine” as situated within a diagnosis and affective formation of collective bodies”* (Ong, 2016; p. 77, emphasis mine).

Given that HLA is being touted as an identity antigen representative of a certain “community” of people, one can say that the social lives and biological materials (Nading, 2017) are conflated in finding an exact match. The risk to health will always remain uncertain (Bharadwaj, 2002), therefore allowing the possibility to capitalize and make a “cultivated cure” (Bharadwaj, 2017) imaginable. Cord blood and genetics are closely linked and redefine personalized therapy for a “community” and the individual. The chance occurrence of falling ill and hoping for a cure in the future in modern healthcare practice gives people the choice of storing their child's cord blood privately, in a community bank or donating it voluntarily.

Biobanking infrastructures can be seen as repositories of information, data, and insurance against stored biosamples. The cord blood unit is processed (personal to the collective in practice) and stored in biobanks for the treatment of blood disorders such as thalassemia. In India, these biobanks holding cord blood are being used to determine stem cell matches where personalized stem cell therapies are sought within social categories. HLA-based stem cell matches in practice include varied definitions of community; in some cases, matches are sought within the family or the extended family, in other cases, caste or race. To complicate the definition of community, matches are sought within the South Asian diaspora or the Tamil-speaking population (collective to the personal in practice), thereby “personalizing” stem cell therapies in Chennai.

## Data availability statement

The raw data supporting the conclusions of this article will be made available by the authors, without undue reservation.

## Ethics statement

The studies involving human participants were reviewed and approved by the Graduate Institute of International and Development Studies, Geneva. The patients/participants provided their written informed consent to participate in this study. Written informed consent was obtained from the individual(s) for the publication of any potentially identifiable images or data included in this article.

## Author contributions

The author confirms being the sole contributor of this work and has approved it for publication.

## Footnotes

^1^“What is a chromosome?” (Medline Plus, 2019).

^2^Medical definition of Mediterranean anemia (MedicineNet, 2022).

^3^The World Marrow Donor Association (WMDA) was set up in 1988, and by 2007, it was established as a worldwide network of blood and marrow transplantation (WBMT). The WBMT is a non-profit scientific organization established to promote stem cell transplantation, donation, and cellular therapy. The WMDA provides a list of guidelines for establishing cord blood banks and registries. Through the stem cell donor registry, the association aims to provide and promote access to stem cells for donors in need across the world (WMDA, 2019).

^4^Screenshots from “Jeevan Be the Cure Thalassemia” (2017)—A short movie made by Be the Cure registry at Jeevan enabling quick, affordable, and free access to matching stem cells from donated cord blood and bone marrow donors to Indians with blood cancers and thalassemia. Available on YouTube: https://www.youtube.com/watch?app=desktop&v=tw_VcPnBiOE&feature=youtu.be.

^5^However, an article by Yang et al. (2006) proved that “Asian” is not a definitive or exclusive category (Robertson, 2012) instead it is mixed, varied (due to migration), and complex. This problematizes the aim of managing a registry based on “Asian” donors.

^6^The *Make in India* initiative was launched by Prime Minister, Narendra Modi, in September 2014 as part of a wider set of nation-building initiatives. Devised to transform India into a global design and manufacturing hub, *Make in India* was both a timely response and opportunistic deflection from a critical situation: by 2013, the much-hyped emerging markets bubble had burst, and India's growth rate had fallen to its lowest level in a decade. The promise of the BRICS Nations (Brazil, Russia, India, China, and South Africa) had faded, and India was tagged as one of the so-called “Fragile Five”. Global investors debated whether the world's largest democracy was a risk or an opportunity. India's 1.2 billion citizens questioned whether India was too big to succeed or too big to fail. India was on the brink of severe economic failure (Make in India, 2019).

^7^About Indian Cells (Indian Cells, 2019).

^8^The Constitution of India under the Seventh Schedule prescribes the Union, State, and Concurrent lists for a division of powers between the Central and State governments. The Union list included subjects such as defense and foreign affairs and laws regarding these are passed by the Parliament. Public health and sanitation, hospitals, and dispensaries fall under the State list, and laws regarding these are passed by the respective state legislatures. Powers to both state and the center are provided in the Concurrent List and include subjects such as education, population control, and family planning. The complete list is available at: The Constitution of India, Ministry of External Affairs (2019).

^9^Writing on blood and nationalism, Robertson (2012) presents evidence that “pure Japaneseness” is qualified and circumscribed by blood. In other words, blood is seen as an active agent of Japanese nationalism and the Japanese started identifying themselves as one national community based on blood types (p. 107).

^10^Referring to Narayan, 2018, human leukocyte antigen (HLA)-A, -B, -C, -DRB1, and -DQB1 haplotype frequencies from 2,491 cord blood units from Tamil-speaking population from Tamil Nadu, India.

^11^Maharashtra is located in central India along the Western coast of India and Marathi is the most commonly spoken language. The government of Maharashtra and the Tata Trust launched a campaign in September 2017 called *Jeev Rakshak Sena* (from Hindi as Life Saving Army) with the aim of creating awareness and opening a bone marrow registry at the Government Medical College and Hospital, Nagpur (The Indian Express, 2017). Maharashtra is part of the sickle cell belt, a point that was stressed upon the launch of the campaign. There is, however, no mention of it being a language-based stem cell donor registry (i.e., Marathi based).

^12^The attempt to consecrate Hindi as the national language was stirred again when a draft of the National Education Policy 2019 by the Modi-led government proposed teaching Hindi and English mandatorily alongside the respective regional language in non-Hindi-speaking states. This draft was later revised to make learning Hindi non-mandatory (Dutta, 2018 & Firstpost, 2018).

^13^Haplotype (National Human Genome Research Institute, 2019).

^14^Phylogeny (n.d.). *Online Merriam-Webster Dictionary*. Retrieved from: https://www.merriam-webster.com/dictionary/phylogeny.
