# Corrigendum: Thalassemia, biobanking infrastructures, and personalized stem cell therapies in Chennai

**DOI:** 10.3389/fsoc.2024.1508436

**Published:** 2024-11-21

**Authors:** Amishi Panwar

**Affiliations:** Department of Population Health Sciences, Bristol Medical School, University of Bristol, Bristol, United Kingdom

**Keywords:** biobanks, Chennai, cord blood, ethnicity, HLA, personalized therapy, thalassemia, stem cells

In the published article, there was an error in the Funding statement. The Funding statement displayed did not acknowledge UKRI/NERC funding during preparation of the article. The correct statement is “The author also acknowledges support from the NERC grant (NE/T013230/1) during preparation of this article.” The correct Funding statement appears below.

